# Responding to HBV and HCV in China and India: thematic series introduction

**DOI:** 10.1186/s41124-016-0023-7

**Published:** 2016-12-15

**Authors:** Phangisile Manciya Mtshali

**Affiliations:** Bristol-Myers Squibb Foundation, Massachusetts, USA

When the World Health Organization (WHO) introduced its first-ever global health sector strategy on viral hepatitis in May 2016, it charged the global community with the goal of eliminating viral hepatitis as a public health threat by 2030. The strategy defines this achievement quantitatively in terms of a number of key targets including a 90% reduction in new chronic hepatitis B virus (HBV) and hepatitis C virus (HCV) infections and a 65% reduction in HBV and HCV deaths by 2030 [1]. The sought-after reductions in incidence and mortality are huge – and at the same time, the strong arsenal of tools at our disposal provides reason for optimism about what can be achieved.

Given the immense burden of HBV and HCV in China and India, however, it simply will not be possible to achieve the WHO targets without taking new measures to meet the challenges facing these two countries. An estimated 5.5% of China’s population of 1.4 billion has the hepatitis B surface antigen (HBsAg). Between 0.2 and 1.2% of the adult population is thought to be HCV RNA-positive [2]. India has a population of 1.2 billion and an estimated HBsAg prevalence level of 1.5% [2], while its estimated adult HCV RNA prevalence level is between 0.4 and 0.8% [3].

The sizes of these countries mean that their disease burdens contribute enormously to global disease burdens. China and India together account for a staggering 37% of all estimated cases of HbsAg infection and 20% of all estimated adult cases of HCV RNA infection worldwide [2, 3].

In this context, the logical question might seem to be: what does the global community need to do to aid India and China? However, the editors of this thematic series on HBV and HCV in India and China would like to reframe the question and urge people working in diverse settings worldwide to consider: what can the world learn from India and China, in terms of how the community-based health programmes of partnerships of government with non-governmental organisations in both countries are responding to complex viral hepatitis-related challenges?

These are, after all, both middle-income countries that have developed policy frameworks for addressing health needs. China has attracted global attention for its activities in the burgeoning field of precision medicine, with large-scale initiatives exploring genetic links to major diseases [4]. In India, a combination of pharmaceutical manufacturing capacity and political will helped with the expansion of the global HIV drug market, supported by technology transfer from and partnership with research and development-based pharmaceutical companies. The more affordable generic alternatives from India have contributed enormously to progress against HIV in low-income countries [5]. Both countries continue to prioritise many important forms of health research through public-sector and private-sector channels and to demonstrate the value of this work to the global community.

Hence my colleagues and I are intrigued by the question of how grassroots and community-based developments in India and China will contribute to progress in the movement to eliminate viral hepatitis, and particularly to ending illness and death from chronic HBV and HCV. The articles in this thematic series provide a fascinating window into the diversity of research activities taking place on the ground in Chinese and Indian communities. In the first article that was published in the series, Chari Cohen and colleagues reported on hepatitis B-related knowledge, attitudes and practices following a citywide public health education program in Haimen City, China, documenting the persistence of stigma in spite of high knowledge levels [6]. This was followed by Hai-Yang Zhou and colleagues’ article on health insurance coverage and medical costs associated with chronic HCV infection in 20 provinces in China [7].

The next article in the series, by Swati Jha and colleagues, reported on findings from a cross-sectional survey assessing HBV knowledge among women of childbearing age in poor communities in Mumbai, India [8]. Subsequently, Ruchi Sogerwal and colleagues published an article that utilized cross-sectional survey data from large cohorts of people who inject drugs in two Indian cities to explore how predisposing, enabling, and need factors might contribute to different levels of HCV testing uptake. Finally, Partha Sarathi Mukherjee and colleagues reported on baseline knowledge levels among newly diagnosed HBV and HCV patients in West Bengal, India. Additionally, a blog post has been published in conjunction with the thematic series, detailing the accomplishments of a large-scale community HBV and HCV educational intervention in Mumbai [9].

In summary, we view this thematic series as an affirmation of the widespread enthusiasm for innovative thinking about HBV and HCV in the Chinese and Indian research communities and community-based program implementers. It is clear that the health crisis brought on by these diseases cannot be resolved entirely through “top-down” interventions by national governments only, and that “bottom-up” approaches are relevant and valuable. They will continue to inform strong national responses. In this regard, we look forward to watching the world learn from China and India in the years to come.

## References

[1] World Health Organization. Draft global health sector strategy on viral hepatitis, 2016-2021: the first of its kind (draft: 13.11.2015) [Internet]. Available from: http://www.who.int/hepatitis/strategy2016-2021/Draft_global_health_sector_strategy_viral_hepatitis_13nov.pdf. Accessed 6 Dec 2016.

[2] Schweitzer A, Horn J, Mikolajczyk RT, Krause G, Ott JJ. Estimations of worldwide prevalence of chronic hepatitis B virus infection: a systematic review of data published between 1965 and 2013. Lancet. 2015; doi: 10.1016/S0140-6736(15)61412-X [Epub ahead of print]. 10.1016/S0140-6736(15)61412-X 26231459

[3] Gower E, Estes C, Blach S, Razavi-Shearer K, Razavi H (2014). Global epidemiology and genotype distribution of the hepatitis C virus infection. J Hepatol.

[4] Cyranoski D (2016). China embraces precision medicine on a massive scale. Nature.

[5] Waning B, Diedrichsen E, Moon S (2010). A lifeline to treatment: the role of Indian generic manufacturers in supplying antiretroviral medicines to developing countries. J Int AIDS Soc.

[6] Cohen C, Evans AA, Huang P, London WT, Block JM, Chen G (2016). Hepatitis B knowledge among key stakeholders in Haimen City, China: Implications for addressing chronic HBV infection. Hepatol Med Policy.

[7] Zhou H, Liu S, Zheng S, Peng X, Chen Y, Duan C (2016). Coverage of different health insurance programs and medical costs associated with chronic hepatitis C infection in mainland China: a cross-sectional survey in 20 provinces. Hepatol Med Policy.

[8] Jha S, Devaliya D, Bergson S, Desai S (2016). Hepatitis B knowledge among women of childbearing age in three slums in Mumbai: a cross-sectional survey. Hepatol Med Policy.

[9] Shukla J, Govale A, Parmar A. A community calling – taking on the viral hepatitis challenge in Mumbai slums [Internet]. http://blogs.biomedcentral.com/on-health/2016/06/14/community-calling-taking-viral-hepatitis-challenge-mumbai-slums/. Accessed 6 Dec 2016.

